# Physical Exercise-Induced Cardiovascular and Thermoregulatory Adjustments Are Impaired in Rats Subjected to Cutaneous Artery Denervation

**DOI:** 10.3389/fphys.2018.00074

**Published:** 2018-02-20

**Authors:** Milene R. Malheiros-Lima, Washington Pires, Ivana A. T. Fonseca, Julliane V. Joviano-Santos, Anderson J. Ferreira, Cândido C. Coimbra, Nilo R. V. Lima, Samuel P. Wanner

**Affiliations:** ^1^Exercise Physiology Laboratory, Department of Physical Education, School of Physical Education, Physiotherapy and Occupational Therapy, Universidade Federal de Minas Gerais, Belo Horizonte, Brazil; ^2^Department of Physical Education, Institute of Life Sciences, Universidade Federal de Juiz de Fora, Governador Valadares, Brazil; ^3^Department of Morphology, Institute of Biological Sciences, Universidade Federal de Minas Gerais, Belo Horizonte, Brazil; ^4^Department of Physiology and Biophysics, Institute of Biological Sciences, Universidade Federal de Minas Gerais, Belo Horizonte, Brazil

**Keywords:** blood pressure, fatigue, physical performance, vascular remodeling, skin, sympathectomy, temperature, thermoregulation

## Abstract

This study aimed to investigate the chronic effects of caudal artery denervation on morphometric parameters of the tail vascular smooth muscle and on physical exercise-induced thermoregulatory and cardiovascular adjustments in rats. Male Wistar rats were subjected to caudal artery denervation or the sham procedure. Approximately 26–28 days after these procedures, their thermoregulatory and cardiovascular parameters were evaluated at rest and during or following a fatiguing treadmill run. At the end of the experiments, the rats were euthanized, and samples of their tails were removed to evaluate morphometric parameters of the vascular smooth muscle surrounding the caudal artery. Denervated rats showed morphological adaptations, including increased arterial wall thickness and wall-to-lumen ratios. In resting rats and following the fatiguing exercise, caudal artery denervation barely affected the thermoregulatory and cardiovascular parameters evaluated. By contrast, caudal artery denervation attenuated the increase in tail skin temperature, decreased the spontaneous baroreflex sensitivity, and exacerbated the increases in mean arterial pressure in exercising rats. The increased wall-to-lumen ratio of denervated rats correlated negatively with the maximum tail skin temperature attained or cutaneous heat loss sensitivity but correlated positively with the maximum diastolic blood pressure attained during exercise. In conclusion, cutaneous denervation induces vascular remodeling characterized by morphological adaptations of the tail vascular smooth muscle. This vascular remodeling likely underlies the impaired tail heat loss and blood pressure adjustments in denervated rats subjected to physical exercise. Therefore, we have highlighted the importance of cutaneous vascular innervation integrity in thermal and cardiovascular control in stress-challenged rats. In this sense, our findings advance the understanding of thermoregulatory and cardiovascular system reactions after a sustained cutaneous vascular innervation injury, which is essential for the treatment of some diseases, such as Parkinson's disease and type 1 and type 2 diabetes mellitus.

## Introduction

The regulation of cutaneous blood flow depends on the thermal and pressure demands of the body, and sympathetic constrictor tone is one of the main regulators of skin vascular resistance (O'Leary et al., 1985; Ootsuka and McAllen, 2006; Ootsuka and Tanaka, 2015). In rats, changes in cutaneous blood flow, particularly in the tail skin vessels, modulate dry heat exchange between the body and the surrounding environment (Rand et al., 1965; Little and Stoner, 1968; Young and Dawson, 1982). The arterio-venous anastomoses connecting the main ventral artery to lateral veins in the proximal portion of the tail effectively carry the blood flow from the body core to the skin surface (Thorington, 1966; Young and Dawson, 1982), thus facilitating heat loss in hyperthermic rats (Vanhoutte et al., 2002). Due to the higher density of these arterio-venous anastomoses, which are associated with a larger surface area-to-mass ratio and a lack of hair, the tail is an important organ for heat loss in passively heated rats or in rats subjected to physical exercise (Wilson et al., 1978; Gordon, 1990; Romanovsky et al., 2002). Under the latter condition, the increase in cutaneous blood flow is an even greater determinant of heat loss because rats cannot spread saliva on their body surface while they are running on a treadmill (Wilson et al., 1978; Shellock and Rubin, 1984). Notably, saliva spreading on the body surface, a behavioral response, facilitates evaporative heat loss in rodents (Hainsworth and Stricker, 1969).

Transection of sympathetic nerves supplying the caudal (tail) artery induces immediate increases in cutaneous blood flow and heat loss (O'Leary et al., 1985; O'Leary and Johnson, 1989; Yanagiya et al., 1999), thereby impairing core body temperature (T_CORE_) homeostasis. Nevertheless, a few days after vascular sympathetic dysfunction, the cutaneous blood flow and heat loss are restored to basal levels (Yanagiya et al., 1999). An augmented sensitivity to vasoconstrictor agents (i.e., catecholamine, vasopressin, and angiotensin II) is currently accepted as a classic adaptive response induced by caudal arterial denervation (CAD) (Webb et al., 1983; Kamikihara et al., 2007; Tripovic et al., 2010), although it is most likely not the sole adaptation. In the rabbit ear, an important thermoregulatory site in this species, adaptations associated with changes in vascular reactivity (Bevan et al., 1993) and vascular remodeling (Bevan, 1975; Bevan and Tsuru, 1981) develop after chronic denervation of the cutaneous artery; this vascular remodeling is characterized by reduced proliferation of vascular smooth muscle (Bevan, 1975) and decreased wall thickness (Bevan and Tsuru, 1981). However, no previous studies have addressed whether morphological adaptations also develop in the vascular smooth muscle surrounding the caudal artery in rats after denervation. Such an investigation is important because different response patterns of endothelium-dependent arterial relaxation have been observed between rats and rabbits (Mackert et al., 1997).

In our previous work, rats subjected to CAD showed a delayed increase in tail skin temperature (T_SKIN_) upon passive exposure to heat on the 26–28th days after denervation (Lima et al., 2013). Therefore, we hypothesized that CAD rats subjected to physical exercise, a physiological condition that increases metabolic heat production, would also exhibit delayed tail vasodilation and cutaneous heat loss. This impaired vasodilation in CAD rats would increase peripheral resistance and place a burden on the cardiovascular system. Additionally, we hypothesized that morphological adaptations in the cutaneous denervated vasculature (if they exist) would be associated with altered cardiovascular and thermoregulatory adjustments during exercise. Therefore, the present study had three objectives: (1) to investigate the exercise-induced thermal and cardiovascular adjustments in CAD rats, (2) to examine the vascular morphological adaptations induced by CAD, and (3) to study the possible associations between these adaptations and changes in thermal and cardiovascular adjustments in exercising rats.

## Materials and methods

### Ethical approval

All procedures were approved by the Ethics Commission for the Care and Use of Animals from the Universidade Federal de Minas Gerais (protocol number 109/09) and were conducted in accordance with the regulations provided by the Brazilian National Council for the Control of Animal Experimentation. Efforts were made to minimize the number of rats used and to minimize their suffering.

### Animals

Adult male *Wistar* rats (*n* = 50, 8 to 10 weeks old at the time of the first surgery) were used in all experiments. Initially, four animals were housed in each standard polypropylene cage. After CAD or sham denervation (Sham-CAD), the rats were housed individually until the end of the experiments. The rats were housed under controlled light (lights on from 05:00 to 19:00 h) and ambient temperature (24.0 ± 1.0°C) conditions. Standard rat chow and tap water were provided *ad libitum*.

### Experimental design

Different groups of rats were subjected to three different sets of experiments, as described below and in Table 1. During all experimental trials, the ambient temperature inside the chamber containing the treadmill belt was maintained at 25°C. After the last experimental trial, the rats were anesthetized (by an intraperitoneal injection of ketamine and xylazine, 116 and 6 mg/kg, respectively), and caudal artery samples were removed for the subsequent histological analysis and verification of the effectiveness of the denervation procedure. The rats were then euthanized by an overdose of anesthesia (i.e., with a dose that was three times higher than that used for anesthesia).

**Table 1:** Surgeries, experimental trials, and parameters recorded for each of the three sets of experiments.

**Set of experiments**	**Surgeries**	**Experimental trials**	**Parameters recorded**
First	Caudal artery denervation	Two incremental-speed exercises: before and on the 26th day after denervation	Time to fatigue and maximum speed attained
Second	Caudal artery denervation and abdominal temperature sensor implant	Resting on the treadmill and constant-speed exercise: on the 26 and 28th day after denervation	Core temperature, tail skin temperature, and time to fatigue
Third	Caudal artery denervation and abdominal temperature sensor and arterial catheter implants	Resting on the treadmill, constant-speed exercise and 30 min of post-exercise: on the 26 and 28th day after denervation	Core temperature, tail skin temperature, pulsatile arterial pressure, and time to fatigue
Post-mortem analyses	Rats taken from the third set of experiments	These analyses followed euthanasia, which was made on the 28th day after denervation	Arterial wall thickness and confirmation of caudal artery denervation

#### First set of experiments: assessment of running performance

Prior to denervation (or sham denervation), the rats were subjected to an incremental-speed exercise to measure their intrinsic aerobic capacity, as determined by the time to fatigue and the maximum speed attained. By matching their body mass and intrinsic aerobic capacity, these rats were divided into two groups, rats subjected to CAD (*n* = 9) and rats subjected to Sham-CAD (*n* = 9). Twenty-six days after these procedures, the rats were once again subjected to an incremental-speed exercise.

#### Second set of experiments: assessment of exercise-induced thermoregulatory responses

The rats were subjected to the CAD (*n* = 10) or Sham-CAD (*n* = 8) procedure. Nineteen days after these procedures (i.e., 1 week before the experimental trials), the rats received an abdominal temperature sensor implant to measure their T_CORE_. Twenty-six days after the CAD or Sham-CAD procedure, the rats were subjected to the first of two experimental trials, which were separated by a 48-h interval: resting on the treadmill or running at a constant speed until volitional fatigue. The order of the trials was balanced and crossed-over. During the experimental trials, T_CORE_, T_SKIN_ (temperature at the lateral surface of the tail), and time to fatigue were measured.

#### Third set of experiments: assessment of exercise-induced cardiovascular responses

Rats were subjected to the CAD (*n* = 8) or Sham-CAD (*n* = 6) procedure. The experimental procedures and measurements were the same as those in the second set of experiments with the exceptions that the pulsatile arterial pressure was measured and physiological responses were recorded for 30 min post-exercise. Twenty-four days after the CAD or Sham-CAD procedure, rats received abdominal temperature sensor implants and arterial catheter implants to measure their T_CORE_ and cardiovascular parameters, respectively. Two days later, the rats were subjected to the first of two experimental trials, which were separated by a 48-h interval.

#### Post-mortem (histological) analyses

Tail slices from 4 Sham-CAD and 6 CAD rats used in the third set of experiments were stored for histological analyses that consisted of morphometric (wall-to-lumen ratio) analyses of the caudal artery. Alternatively, the caudal artery sections from the other rats were used to confirm the effectiveness of denervation.

### General surgical procedures

For all surgical procedures, rats were anesthetized with an intraperitoneal injection of a cocktail containing ketamine and xylazine (116 and 6 mg/kg, respectively). After anesthesia induction, the rats were treated with prophylactic doses of different antibiotics (including 48.000 IU/kg intramuscular benzylpenicillin). A post-operative analgesic (1.1 mg/kg subcutaneous flunixin meglumine) was also administered.

#### Caudal artery denervation

The procedure used for caudal artery denervation was described previously (Lima et al., 2013). Briefly, the ventral tail artery was exposed by two incisions ~8 cm in length. The first and second incisions were made in the skin and subcutaneous tissue, respectively, with both incisions starting from the base of the tail. The proximal portion of the ventral tail artery was gently dissected, and all surrounding tissues were removed, taking care to preserve the arteriovenous anastomoses. Then, the surface of the tail artery was painted with a topical phenol solution (10%, diluted in glycerol) to eliminate local vascular innervation. The Sham-CAD rats were subjected to only the 8-cm ventral incision in the tail skin, which was then sutured; this sham procedure did not compromise any innervation to the tail artery (Lima et al., 2013).

The innervation of the distal portion of the caudal artery was intentionally left intact in our study, as evidence suggests that only the proximal portion contributes to thermoregulation. Most heat exchange and vasoconstriction occur in the proximal portion (a few cm from the tail base); thus, the distal portion is largely poikilothermic and mimics the environmental temperature (Thorington, 1966). The poikilothermic characteristic of the distal portion was also examined in an experimental paradigm consisting of isolated perfused rat tail, where the tail skin temperature in the distal portion was similar to the ambient temperature (Redfern et al., 1995). In addition, the magnitude of heat-induced vasodilation, as estimated by measuring the arterial-venous temperature difference using pairs of surface temperature probes, decreased at more distal positions along the tail (Young and Dawson, 1982).

#### Temperature sensor implant

A telemetry transmitter sensor (TR3000 XM-FM model, Mini-Mitter, Bend, OR, USA) was inserted into the abdominal cavity via two small (~2 cm in length) incisions in the abdominal skin and in the *linea alba* of the rectus abdominis. Following insertion, the abdominal muscle and skin were sutured in layers.

#### Arterial catheter insertion

The left common carotid artery was identified through a ventral incision in the neck, and its proximal portion was occluded. A small cut was made in the artery, thereby allowing for the insertion of a polyethylene catheter (PE-10 connected to a PE-50; Becton Dickinson, Franklin Lakes, NJ, USA) filled with heparin diluted in isotonic saline. The free end (PE-50) of the polyethylene tubing was tunneled subcutaneously and exteriorized at the cervical dorsal area (Pires et al., 2010, 2013).

### Measurements and calculations

#### Thermoregulatory parameters

T_CORE_ was measured by telemetry using a temperature sensor implanted in the abdominal cavity. This telemetric sensor sent pulses at different frequencies according to the T_CORE_ value. The radio wave frequency was captured by a receiver plate (Mini-Mitter) positioned next to the treadmill. T_SKIN_ was measured using a thermocouple fixed to the right lateral surface (or ventral surface) located 1 cm from the base of the tail. The close proximity to the base of the tail enabled more sensitive measurements of changes in T_SKIN_ that occurred as a function of changes in local blood flow (Young and Dawson, 1982). Notably, the T_SKIN_ in heat-exchange organs, such as the rat tail, primarily depends on the local vasomotor tone and is not a reliable measure of ambient temperature (Romanovsky, 2014). The ambient temperature (T_A_) was measured by a thermocouple taped to the ceiling of the treadmill chamber located close to the stimulating grid at the rear end of the treadmill belt.

#### Variability in body temperature

We determined the deviation of each temperature measurement (T_CORE_ or T_SKIN_) from the average value obtained throughout the resting experiments to analyze the variability in T_CORE_ and T_SKIN_. Next, all deviation values were averaged for each animal and then averaged for each group.

#### Threshold and sensitivity of cutaneous heat loss

T_SKIN_ was plotted against T_CORE_ for each animal during exercise to characterize the differences in cutaneous heat loss between the experimental groups. The approximate location of the T_SKIN_ threshold was identified visually, and experimental data before and after this threshold were separated. A linear regression analysis was then performed to describe the relationship between T_SKIN_ and T_CORE_, and the intersection of the regression lines (before and after) was used to determine the heat loss threshold (HL_THR_), which represents T_CORE_ at the initiation of the rapid increase in T_SKIN_. Heat loss sensitivity (HL_SEN_) was defined as the regression slope of the five time points that followed the threshold and corresponded to the steepest part of the rising curve. This analysis has been used in recent studies with exercising rats to determine whether mechanisms underlying modified cutaneous heat loss have a central and/or peripheral origin (Wanner et al., 2015a; Drummond et al., 2016). In this context, changes in the onset threshold are traditionally considered indicators of central modulation (Gisolfi and Wenger, 1984), whereas changes in sensitivity often describe peripheral adaptations in thermoeffector responses (Sawka et al., 2011).

#### Cardiovascular parameters

Heart rate (HR), systolic arterial pressure (SAP), diastolic arterial pressure (DAP), and mean arterial pressure (MAP) were determined from pulsatile arterial pressure recordings using AcqKnowledge 3.7.0 software (Biopac Systems, Goleta, CA, USA).

#### Heart rate and blood pressure variability

The tape-recorded arterial pressure signal was sampled at 2 kHz. The SAP values were identified beat by beat, and the pulse interval was calculated as the interval between two consecutive systolic peaks using a customized routine (MATLAB 7.8, MathWorks, Natick, MA, USA). Time- and frequency-domain analyses were performed during resting, constant-speed exercise and after exercise using an 8-min period selected from continuous recordings after stabilization of the cardiovascular parameters. In exercising rats, the middle point of the 8-min recording corresponded to half of the time to fatigue. The power spectral density was obtained with a fast Fourier transformation, and the size of the evaluated segment was established using 512 points with 50% overlap. The spectral power for very low frequency ([VLF] < 0.2 Hz), low frequency ([LF] = 0.2–0.75 Hz), and high frequency ([HF] > 0.75 Hz) bands was evaluated. The LF-to-HF ratio of pulse interval variability was calculated to assess the sympatho-vagal balance in the heart (Montano et al., 1994). These bandwidths have previously been used to analyze the blood pressure spectrum and HR variability in rats (Ceroni et al., 2009; Tezini et al., 2013; Müller-Ribeiro et al., 2017). Integration of the power spectrum density within each frequency bandwidth was achieved using Cardioseries software (version 2.2; Ribeirão Preto, SP, Brazil).

#### Spontaneous baroreflex sensitivity

The baroreflex gain was determined from spontaneous changes in blood pressure and the pulse interval using a time-series method designed for the rat (Oosting et al., 1997; Waki et al., 2006; Müller-Ribeiro et al., 2017). First, spontaneously occurring ramps of either decreasing or increasing blood pressure of four beats were used to calculate the baroreceptor reflex sensitivity. Second, for each pair of blood pressure and pulse interval ramps, measurements were collected at delays of three, four and five beats based on the delay time from a change in blood pressure to a reflex response in the pulse interval in the rat, as described by Oosting et al. (1997). Third, from these three ramps, plots of the changes in pulse interval vs. blood pressure were generated to calculate the slopes of each of the delays, and average values for the slope were calculated for each of the delays. Finally, the calculated baroreflex sensitivity value represents the mean value of the three values (i.e., delays) from ramps with positive or negative slopes.

### Exercise sessions

#### Familiarization with running on a treadmill

Rats used in the three sets of experiments were familiarized with running on a treadmill, and this protocol was always initiated 5 days prior to the first experimental trial. The familiarization protocol consisted of a daily exercise session, and the rats were gradually encouraged to exercise on a treadmill designed for small animals (Modular Treadmill, Columbus Instruments, Columbus, OH, USA) using light electrical stimulation (0.5 mA). In each session, the rats ran at a constant speed of 18 m·min^−1^ and at an incline of 5% for 5 min (Wanner et al., 2007; Guimarães et al., 2013). The same treadmill incline and level of electrical stimulation used in the familiarization sessions were used during the experimental trials (i.e., incremental- and constant-speed treadmill running).

#### Incremental-speed treadmill running

Exercise was initiated at a speed of 10 m·min^−1^, and this speed was increased by 1 m·min^−1^ every 2 min until the rats were fatigued. Fatigue was defined as the moment when the rats could no longer maintain the running pace and exposed themselves to the light electrical stimulation for 10 s (Wanner et al., 2007; Pires et al., 2013).

#### Running performance

Performance was evaluated during incremental-speed running by determining the time to fatigue (min) and the maximum speed (S_MAX_) attained by the rats. During constant-speed running, performance was evaluated by determining the time to fatigue (min). The S_MAX_ attained during the incremental exercise sessions was calculated by modifying the following equation proposed by Kuipers et al. (1985) for calculating maximal power output: S_MAX_ = S1 + [S2(t/120)], in which S1 is the speed reached in the last completed stage (m.min^−1^), S2 is the increment in treadmill speed at each stage (m.min^−1^), and t is the time spent in the uncompleted stage (s).

#### Constant-speed treadmill running

The treadmill speed was maintained at a constant value of 18 m·min^−1^, which corresponded to ~70% of the maximum speed attained by rats of both groups during incremental running (please see **Figure 3C**). The constant exercise bout was interrupted when rats were fatigued (as defined using the same criterion for incremental running). In the third set of experiments, the thermoregulatory and cardiovascular parameters continued to be recorded for an additional 30 min after the end of the exercise to evaluate whether CAD impaired physiological responses during the post-exercise period.

#### Resting

When the cardiovascular and thermoregulatory parameters were stable for at least 20 min (usually 2–4 h after the rats had been placed on the treadmill), the resting values for these parameters were recorded over the next 45 min.

### Histology and immunohistochemistry evaluations

Before removing tail samples, rats were anesthetized and successively transcardially perfused with a saline solution and paraformaldehyde (4% in 0.1 M phosphate buffer, pH = 7.4). The tail samples were stored in 70% ethanol prior to starting the decalcification procedure. After decalcification, tissue samples were dehydrated in a graded series of ethanol (70, 95, and 100%), embedded in resin (2-hydroxymethacrylate), and polymerized at 56°C. The paraffin blocks were cut (10 μm), and tail slices were placed on slides. The slides were stained with hematoxylin and eosin and then coverslipped with entellan.

#### Morphometric analyses

Photomicrographs of transverse sections of the caudal artery were acquired (10-fold increase), digitized and analyzed off-line using ImageJ software. The vascular and lumen perimeters, which correspond to the contour of the vascular and lumen areas, respectively, were determined by the software after initial calibration. The vascular and lumen diameters were also determined by the software; in this case, four different measurements were collected for each rat and averaged. The arterial wall thickness was calculated by subtracting the inner diameter from the outer diameter and then dividing the result by two. Furthermore, the wall-to-lumen ratio was calculated to characterize the presence of vascular remodeling (Melo et al., 2003).

#### Glyoxylic acid fluorescence histochemical analysis

The tail artery was cut into 10-μM rings using a cryostat microtome at −30°C, and the rings were mounted in slices and then immersed three times (30 s each) in a 2% glyoxylic acid solution (diluted in 0.1 M phosphate buffer adjusted to pH 7.4 with 10 M NaOH). The slices were dried (for 20 min in a stream of cold air) and covered with mineral oil before being heated at 60°C for 30 min. Next, the slices were coverslipped and visualized under a fluorescence microscope. Catecholaminergic innervation of the caudal arteries was visualized and photographed using a microscope equipped with an HBO 100-W mercury light source (Axioplan-Zeiss Microscope, Carl Zeiss, Oberkochen, Germany). The effectiveness of the caudal artery denervation procedure was confirmed by the absence of catecholamine fluorescence in caudal artery slices (Lima et al., 2013).

### Statistical analysis

Data normality was assessed using the Shapiro-Wilk test, and all normally distributed data are expressed as the means ± standard errors of the means (SE). The T_SKIN_, T_CORE_, HR, and MAP data were compared between groups (i.e., Sham-CAD vs. CAD rats) and across time points using two-way ANOVAs with repeated measures analysis for only the time factor. When applicable, Tukey's *post-hoc* test was used. Inter-group differences in body temperature variability, HL_THR_, HL_SEN_, HR and SAP variability, spontaneous baroreflex sensitivity, body mass, time to fatigue, maximum speed attained, and morphometric parameters were evaluated using unpaired Student's *t*-tests. The correlations presented in the results were assessed using the Pearson coefficient. α < 0.05 was considered statistically significant.

## Results

### Effectiveness of caudal artery denervation

**Figure 1:**
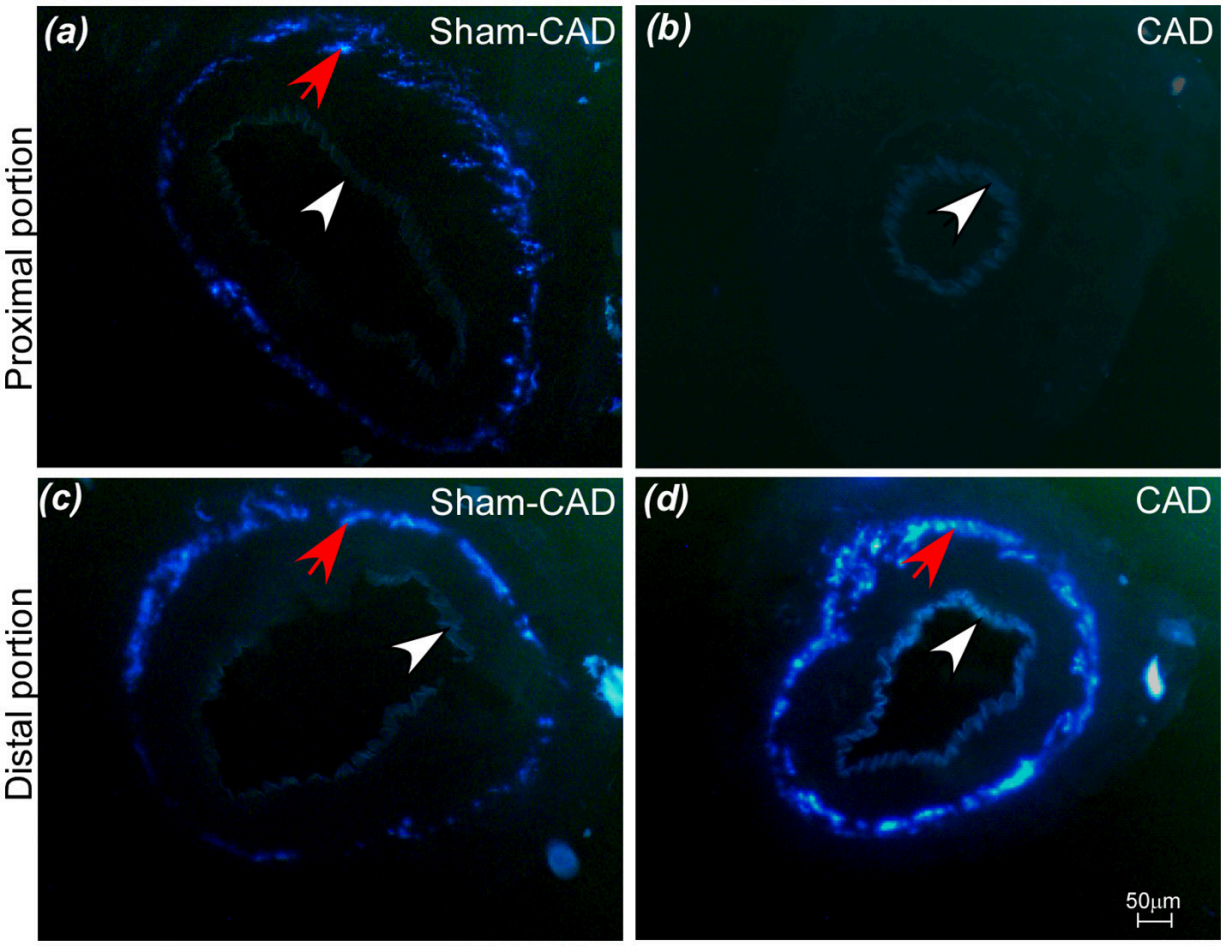
Effectiveness of the denervation procedure. Denervation was confirmed by the absence of endogenous catecholamine-mediated fluorescence at the adventitial-medial junction in transverse sections (**A** Sham-CAD rat and **B** CAD rat) of a proximal portion of the caudal artery. The denervation procedure was restrained to the area where phenol was applied, as evidenced by the presence of fluorescence at the adventitial-medial junction in transverse sections of a distal portion of the caudal artery (**C** Sham-CAD rat and **D** CAD rat). The red arrows indicate the presence of sympathetic innervation of the vascular smooth muscle, whereas the white arrows indicate autofluorescence of the internal elastic lamina.

Fluorescence induced by glyoxylic acid was evaluated 26–28 days after the CAD or Sham-CAD surgery (Figure 1) and was used to confirm the effectiveness of denervation and verify that reinnervation did not occur. The high density of sympathetic innervation in the adventitial-medial junction of the caudal artery, as indicated by endogenous catecholamine-mediated fluorescence in transverse slices of the artery in Sham-CAD rats (red arrows in Figures 1A,C), was completely abolished in CAD rats (Figure 1B) after phenol application. Moreover, only the vascular portion subjected to phenol application (i.e., the proximal portion) was indeed denervated; the distal portion of the caudal artery (Figure 1D), a non-essential region for cutaneous heat exchange with the surroundings (Thorington, 1966; Young and Dawson, 1982; Redfern et al., 1995), was not affected by chemical neurolysis.

### Thermoregulatory and cardiovascular parameters in resting rats

**Figure 2:**
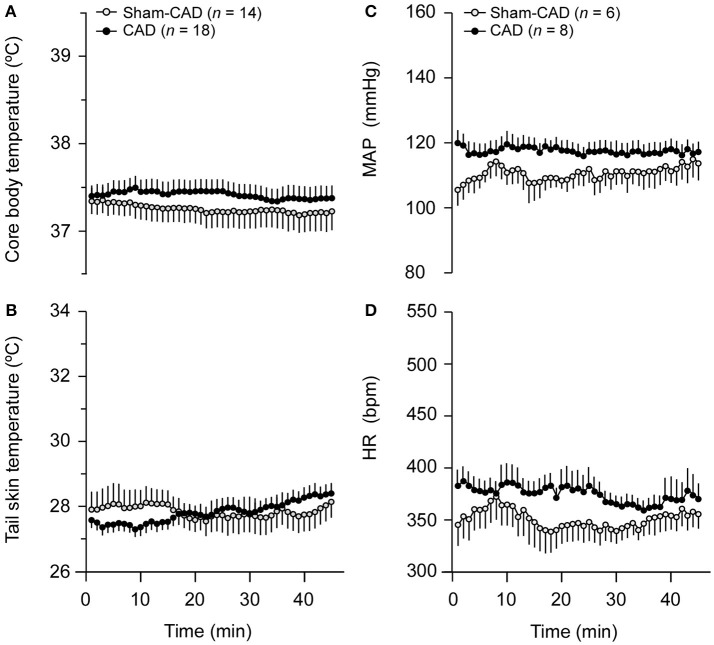
Effects of caudal artery denervation (CAD) on thermoregulatory and cardiovascular responses in resting rats. Thermoregulatory responses comprise the core body temperature **(A)** and tail skin temperature **(B)**, whereas cardiovascular responses comprise the mean arterial pressure (MAP; **C**) and heart rate (HR; **D**). Values are expressed as the means ± standard errors (SE).

When allowed to rest on the treadmill belt, the control rats showed the expected values for T_CORE_, T_SKIN_, MAP, and HR (Figure 2). The heat loss index of these control rats was ~0.2. Based on a previously developed method (Romanovsky et al., 2002), the heat loss index data suggest that the environmental conditions corresponded to the lower extremity of the thermoneutral zone (Lima et al., 2013; Wanner et al., 2015b). As expected, CAD rats did not show differences in their resting T_CORE_ and T_SKIN_ (Figures 2A,B) but showed lower T_SKIN_ variability (0.63 ± 0.06°C vs. 0.86 ± 0.07°C; *p* = 0.005) and higher T_CORE_ variability (0.22 ± 0.03°C vs. 0.17 ± 0.01°C; *p* = 0.029) than the control rats. Regarding the cardiovascular responses (Figures 2C,D), CAD rats tended to have higher MAP (118 ± 3 vs. 111 ± 5 mmHg; *p* = 0.097, average values for the 45-min recordings) and HR values than the Sham-CAD rats (375 ±10 vs. 351 ± 14 bpm; *p* = 0.119, average values). Next, we evaluated the autonomic control of the cardiovascular system by performing a spectral analysis of the HR and SAP recordings (Table 2). Neither the SAP nor the HR variability parameters were affected by CAD. Additionally, the spontaneous baroreflex sensitivity did not differ between the two groups of resting rats (Table 2).

**Table 2:** Autonomic control of the cardiovascular system in resting rats subjected to caudal artery denervation or the sham procedure.

	**Sham-CAD (*n* = 5)**	**CAD (*n* = 5)**	***p*-value**
**HEART RATE**			
Pulse interval (ms)	167 ± 4	172 ± 4	0.391
SD (ms)	3.9 ± 0.1	3.3 ± 0.3	0.179
Variance (ms^2^)	14.8 ± 0.4	11.5 ± 1.8	0.158
RMSSD (ms)	0.4 ± 0.04	0.4 ± 0.02	0.774
LF (mmHg^2^)	0.13 ± 0.04	0.06 ± 0.01	0.179
HF (ms^2^)	0.07 ± 0.02	0.06 ± 0.01	0.856
LF-to-HF ratio	1.7 ± 0.4	0.9 ± 0.1	0.091
**SYSTOLIC ARTERIAL PRESSURE**			
Systolic arterial pressure (mmHg)	126 ± 3	127 ± 5	0.968
SD (mmHg)	3.6 ± 0.4	3.3 ± 0.4	0.609
Variance (mmHg^2^)	13.5 ± 2.6	11.5 ± 2.6	0.616
LF (mmHg^2^)	0.61 ± 0.17	0.72 ± 0.12	0.650
HF (mmHg^2^)	0.05 ± 0.03	0.02 ± 0.01	0.266
**SPONTANEOUS BAROREFLEX SENSITIVITY**			
Gain (ms·mmHg^−1^)	1.35 ± 0.06	1.30 ± 0.05	0.498

*CAD, rats subjected to caudal artery denervation; HF, high frequency; LF, low frequency; RMSSD, root mean square of successive differences; SD, standard deviation; Sham-CAD, rats subjected to the sham surgery. Values are expressed as the means ± SE*.

### Running performance as measured using two different exercise protocols

**Figure 3:**
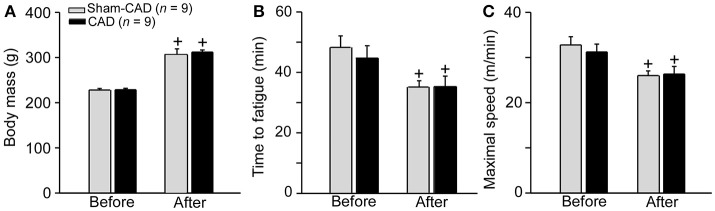
Effects of caudal artery denervation (CAD) on body mass **(A)** and physical performance, expressed as the time to fatigue **(B)** or maximum speed **(C)**, of rats subjected to incremental-speed exercises before and 26 days after the denervation or sham-denervation procedure. Values are expressed as the means ± SE. + Significantly different (*p* < 0.05) from the “before” measurements in the same group of rats.

We initially subjected the rats to an incremental-speed running exercise that was performed before (intact rats) and 26 days after the denervation or sham procedure to investigate whether CAD changed the rats' physical performances. We selected this exercise protocol because a previous study showed that the variation coefficient of the time to fatigue was lower during incremental-speed running than during constant-speed running (Pires et al., 2013). Compared to the results of the first test (intact group), the results of the second test indicated reductions in the time to fatigue and maximal speed (in both the Sham-CAD and CAD groups) (Figures 3B,C). Moreover, inter-group differences were not observed in the second test (35 ± 2 vs. 35 ± 3 min; *p* = 0.967). During the 26-day period between surgery and the second test, the body masses of rats from both groups increased by ~80 g with no inter-group differences (Figure 3A). A significant negative correlation was observed between the time to fatigue and body mass (*r* = −0.420; *r*^2^ = 0.176; *p* = 0.010).

The exercise intensity used during constant-speed running corresponded to ~70% of the maximum speed attained by the rats (Sham-CAD: 69 ± 18%; CAD: 68 ± 11%; *p* = 0.933) (Figure 3C). As expected, no inter-group differences were observed in the time to fatigue during constant-speed running (Sham-CAD: 39 ± 3 min; CAD: 41 ± 4 min; *p* = 0.773).

### Thermoregulatory and cardiovascular control parameters in running rats

**Figure 4:**
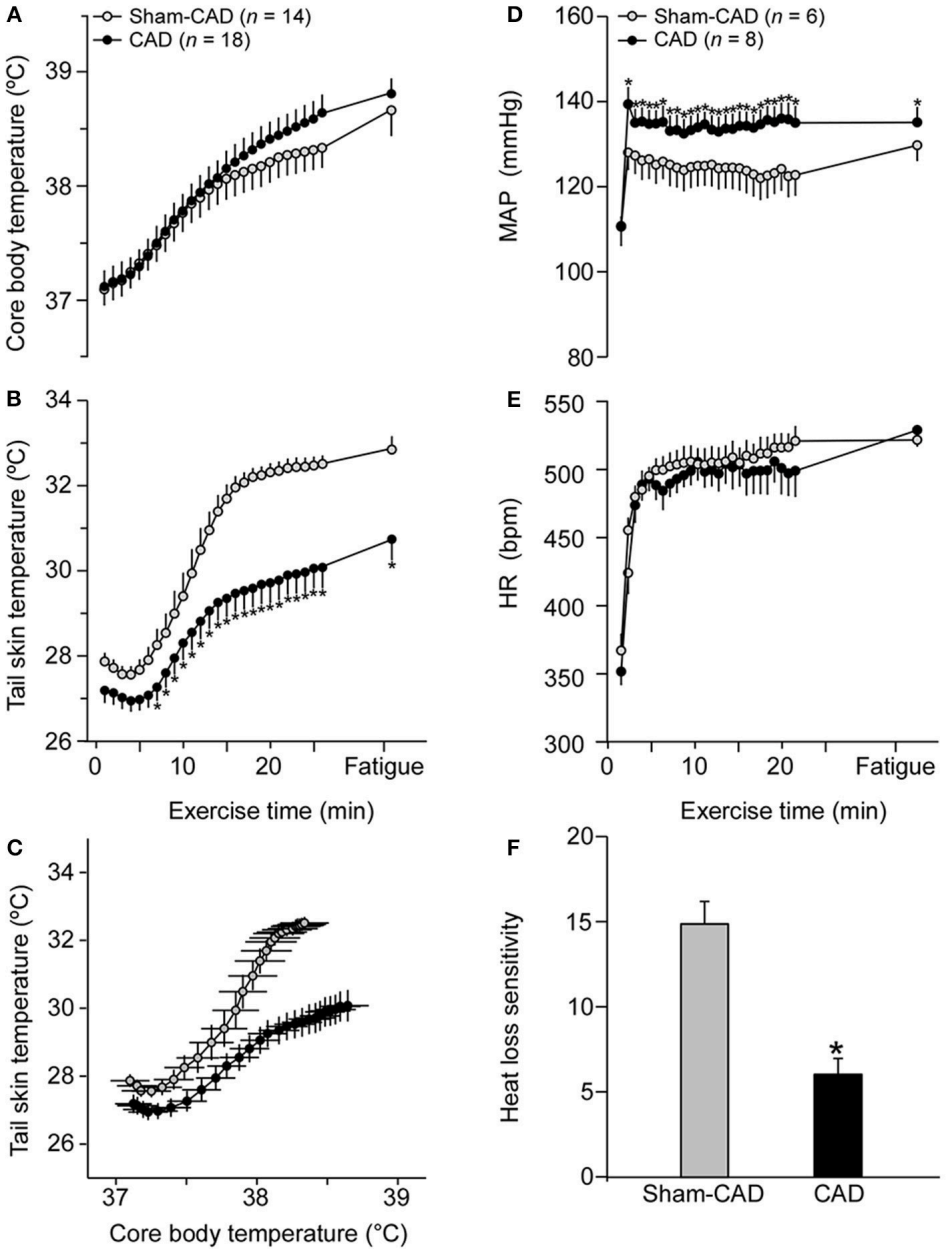
Effects of caudal artery denervation (CAD) on thermoregulatory and cardiovascular responses induced by constant-speed exercise. The thermoregulatory responses comprise the core body temperature **(A)**, tail skin temperature **(B)**, tail skin temperature plotted against core body temperature **(C)**, and sensitivity of cutaneous heat loss **(F)**, whereas the cardiovascular responses comprise the mean arterial pressure (MAP; **D**) and heart rate (HR; **E**). Values are expressed as the means ± SE. ^*^Significantly different (*p* < 0.05) from the Sham-CAD rats.

The constant-speed exercise increased T_CORE_ and T_SKIN_ of the control rats by 1.5 and 5.0°C, respectively. Regarding the T_CORE_ response, two-way ANOVA revealed an interaction between the groups and exercise time points (*F* = 3.48; *p* < 0.001); however, *post-hoc* analysis did not identify any differences in means between CAD and Sham-CAD rats during treadmill running (Figure 4A), including measurements collected at fatigue (CAD: 38.81 ± 0.12°C vs. Sham-CAD: 38.67 ± 0.22°C; *p* = 0.166). Additionally, CAD attenuated the exercise-induced increase in T_SKIN_, which was ~2°C lower in CAD rats than in Sham-CAD rats at fatigue (Figure 4B). CAD rats also displayed lower HL_SEN_ (6.02 ± 0.94 vs. 14.91 ± 1.32; *p* < 0.001; Figures 4C,F), but not HL_THR_ (CAD: 37.53 ± 0.15°C vs. Sham-CAD: 37.66 ± 0.20°C; *p* = 0.585), than Sham-CAD rats (Figure 4C).

Physical exercise also increased MAP and HR in Sham-CAD rats. The tail artery denervation procedure exacerbated the increases in MAP (Figure 4D), SAP and DAP (*data not shown*) without affecting the increase in HR (Figure 4E). Regarding the autonomic control of the cardiovascular system, denervation did not change the components of HR variability but increased the LF component of SAP variability (Table 3). Finally, the spontaneous baroreflex sensitivity was reduced in CAD rats compared to that in Sham-CAD rats (Table 3).

**Table 3:** Autonomic control of the cardiovascular system in exercising rats subjected to caudal artery denervation or the sham procedure.

	**Sham-CAD (*n* = 6)**	**CAD (*n* = 8)**	***p*-value**
**HEART RATE**			
Pulse interval (ms)	119, 5	117, 3	0.754
SD (ms)	1.8, 0.4	1.8, 0.2	0.874
Variance (ms^2^)	4.1, 1.8	3.5, 0.8	0.727
RMSSD (ms)	0.4, 0.04	0.4, 0.02	0.251
LF (mmHg^2^)	0.21, 0.05	0.36, 0.09	0.238
HF (ms^2^)	0.19, 0.01	0.27, 0.01	0.231
LF-to-HF ratio	1.0, 0.1	1.3, 0.2	0.353
**SYSTOLIC ARTERIAL PRESSURE**			
Systolic arterial pressure (mmHg)	136, 4	147, 3^*^	0.034
SD (mmHg)	3.0, 0.2	3.4, 0.1	0.114
Variance (mmHg^2^)	9.1, 1.1	11.5, 1.0	0.132
LF (mmHg^2^)	1.76, 0.48	2.93, 0.40^*^	0.046
HF (mmHg^2^)	0.18, 0.04	0.20, 0.03	0.287
**SPONTANEOUS BAROREFLEX SENSITIVITY**			
Gain (ms·mmHg^−1^)	1.41, 0.13	1.21, 0.08^*^	0.043

CAD, rats subjected to caudal artery denervation; HF, high frequency; LF, low frequency; RMSSD, root mean square of successive differences; SD, standard deviation; Sham-CAD, rats subjected to the sham surgery;

* *significantly different (p < 0.05) from the Sham-CAD rats. Values are expressed as the means ± SE*.

### Post-exercise thermoregulatory and cardiovascular parameters in rats

**Figure 5:**
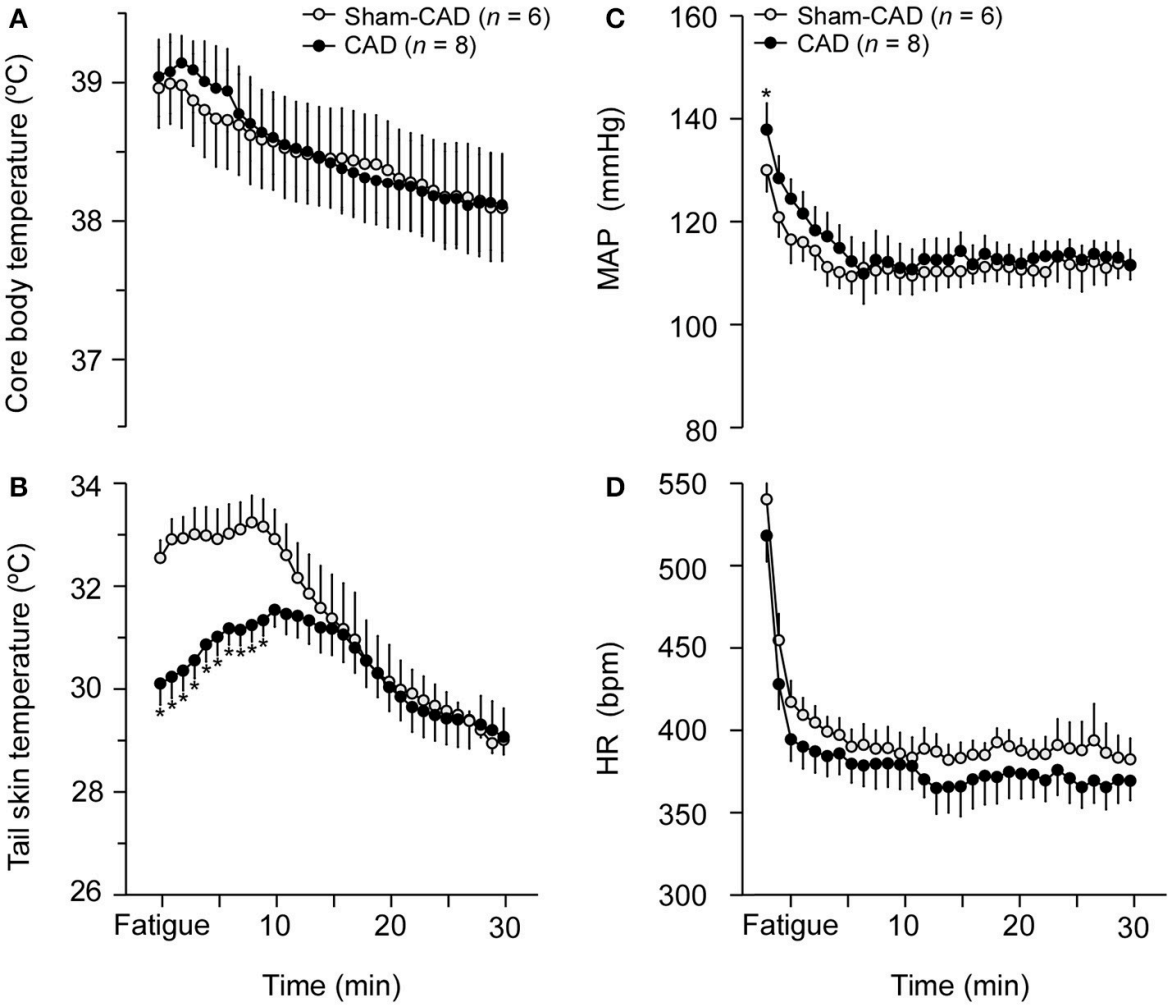
Effects of caudal artery denervation (CAD) on thermoregulatory and cardiovascular responses during the post-exercise period. The thermoregulatory responses comprise the core body temperature **(A)** and tail skin temperature **(B)**, whereas the cardiovascular responses comprise the mean arterial pressure (MAP; **C**) and heart rate (HR; **D**). Values are expressed as the means ± SE. ^*^Significantly different (*p* < 0.05) from the Sham-CAD rats.

After exercise cessation, T_CORE_ gradually decreased in the control rats, and this response was not affected by CAD. At the end of the post-exercise period (30 min), T_CORE_ was still higher than the resting values of both groups (Figure 5A). Regarding cutaneous heat loss, T_SKIN_ remained constant in the control rats for 10 min and then gradually decreased until it reached values similar to those at rest (Figure 5B). In contrast, T_SKIN_ increased in CAD rats for the first 10 min, attained similar values to the control group and then gradually decreased toward the resting values. Therefore, when comparing the kinetics of T_SKIN_ during the initial 10 min of the post-exercise period, CAD rats exhibited greater increases in this temperature than Sham-CAD rats.

During the post-exercise period, MAP and HR were initially markedly reduced in control rats, followed by a prolonged and slower reduction (Figures 5C,D). CAD did not change the post-exercise kinetics (i.e., reduction) of these parameters. We then evaluated the autonomic control of the cardiovascular system, and none of the parameters associated with HR and SAP variability differed between the two groups (Table 4).

**Table 4:** Autonomic control of the cardiovascular system during the post-exercise period in rats subjected to caudal artery denervation or the sham procedure.

	**Sham-CAD (*n* = 6)**	**CAD (*n* = 8)**	***p*-value**
**HEART RATE**			
Pulse interval (ms)	159 ± 4	164 ± 8	0.619
SD (ms)	3.2 ± 0.3	3.1 ± 0.2	0.657
Variance (ms^2^)	10.7 ± 1.6	9.8 ± 1.2	0.631
RMSSD (ms)	0.50 ± 0.05	0.43 ± 0.02	0.254
LF (mmHg^2^)	0.11 ± 0.03	0.12 ± 0.02	0.862
HF (ms^2^)	0.10 ± 0.04	0.07 ± 0.01	0.367
LF-to-HF ratio	1.49 ± 0.54	1.65 ± 0.15	0.769
**SYSTOLIC ARTERIAL PRESSURE**			
Systolic arterial pressure (mmHg)	122 ± 1	126 ± 4	0.325
SD (mmHg)	3.2 ± 0.4	3.2 ± 0.4	0.892
Variance (mmHg^2^)	10.6 ± 2.0	11.2 ± 3.0	0.858
LF (mmHg^2^)	0.80 ± 0.42	0.86 ± 0.26	0.905
HF (mmHg^2^)	0.02 ± 0.01	0.09 ± 0.05	0.203
**SPONTANEOUS BAROREFLEX SENSITIVITY**			
Gain (ms·mmHg^−1^)	1.43 ± 0.18	1.18 ± 0.15	0.183

*CAD, rats subjected to caudal artery denervation; HF, high frequency; LF, low frequency; RMSSD, root mean square of successive differences; SD, standard deviation; Sham-CAD, rats subjected to the sham surgery. Values are expressed as the means ± SE*.

### Histological evaluation after denervation

**Figure 6:**
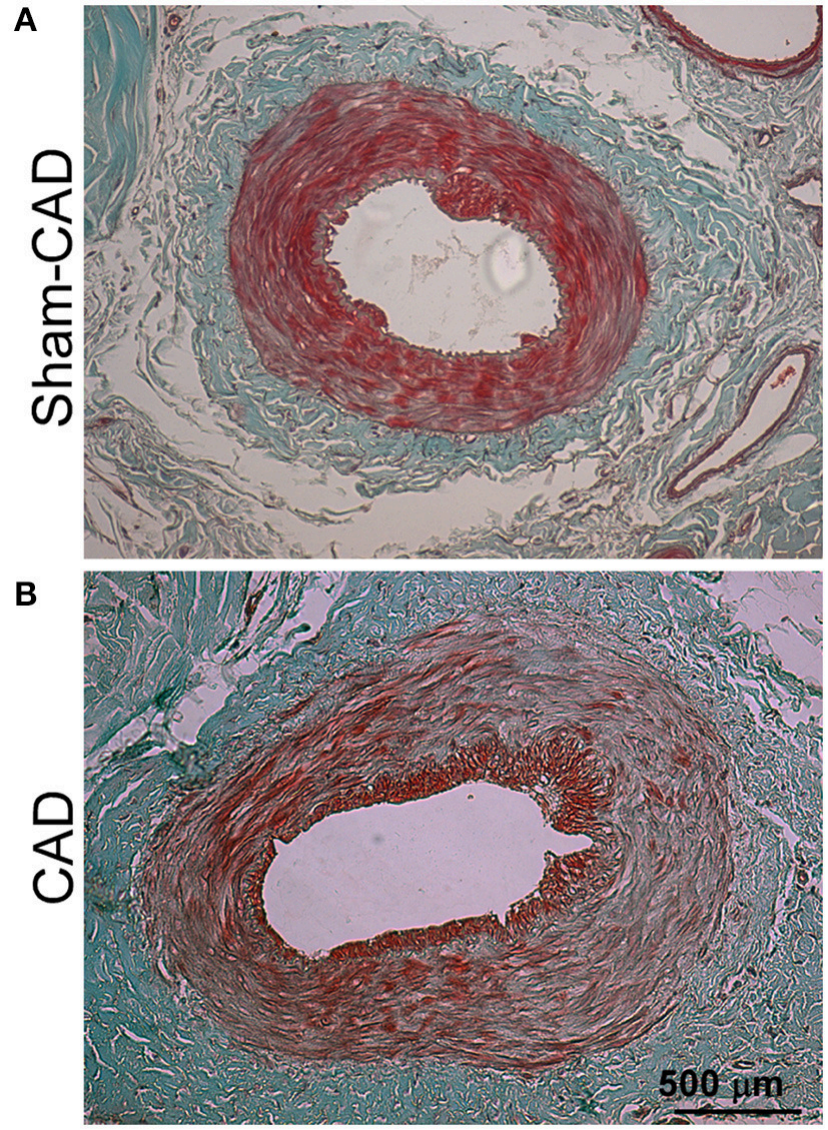
Effects of caudal artery denervation (CAD) on the morphometry of the tail artery. **(A,B)** show representative photomicrographs of the proximal portions of the tails of Sham-CAD and CAD rats stained with hematoxylin and eosin.

Caudal artery denervation increased the arterial wall thickness and wall-to-lumen ratio in rats, as illustrated in Figure 6 and Table 5. Furthermore, the vascular diameter and perimeter were also greater in CAD rats than in Sham-CAD rats. In contrast, no inter-group differences were observed in the lumen diameter and perimeter (Table 5). Collectively, this morphometric analysis indicates that CAD induces vascular remodeling.

**Table 5:** Morphometric analyses of the caudal (tail) artery in rats subjected to caudal artery denervation or the sham procedure.

**Parameters**	**Sham-CAD (*n* = 4)**	**CAD (*n* = 6)**	***p*-value**
Wall-to-lumen ratio	0.53 ± 0.04	0.88 ± 0.08^*^	0.008
Arterial wall thickness (μm)	305 ± 13	414 ± 31^*^	0.007
Vascular perimeter (μm)	3891 ± 225	4861 ± 270^*^	0.014
Lumen perimeter (μm)	2209 ± 260	2268 ± 187	0.848
Vascular diameter (μm)	1236 ± 56	1444 ± 51^*^	0.025
Lumen diameter (μm)	626 ± 41	616 ± 37	0.886

CAD, rats subjected to caudal artery denervation; Sham-CAD, rats subjected to the sham surgery;

* *significantly different (p < 0.05) from the Sham-CAD rats. Values are expressed as the means ± SE*.

### Correlation between artery denervation-induced changes in arterial thickness and cutaneous heat loss, HL_SEN_, or DAP

**Figure 7:**
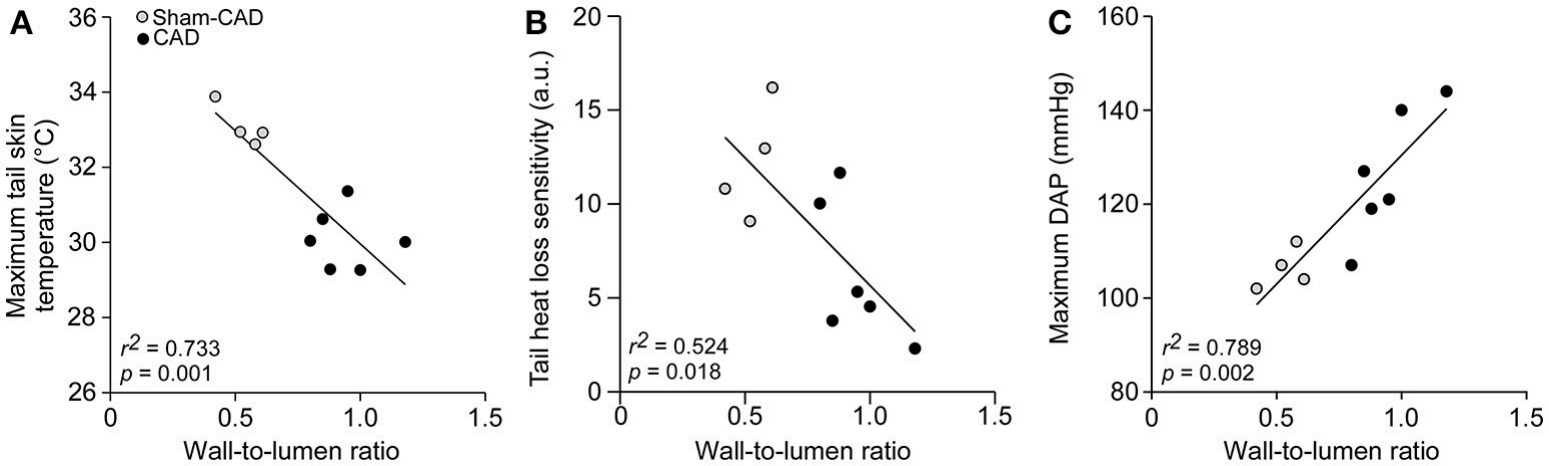
Correlations between the wall-to-lumen ratio and the maximum tail skin temperature attained during exercise **(A)**, tail heat loss sensitivity **(B)**, and maximum diastolic arterial pressure attained during exercise **(C)**.

Pearson's correlation coefficients were used to evaluate the strength of the association between CAD-mediated physiological changes during exercise and the CAD-mediated increase in the wall-to-lumen ratio. As shown in Figure 7, significant negative correlations were observed between the maximum T_SKIN_ attained (*r* = −0.856; *r*^2^ = 0.733; *p* = 0.001) or HL_SEN_ (*r* = −0.724; *r*^2^ = 0.524; *p* = 0.018) during exercise and the wall-to-lumen ratio. We also observed a positive correlation between the maximum DAP attained during treadmill running and the wall-to-lumen ratio (*r* = 0.888; *r*^2^ = 0.789; *p* = 0.002).

## Discussion

The present study investigated the chronic effects of CAD (26–28 days after nerve lesion) on thermoregulatory and cardiovascular adjustments in rats subjected to treadmill running. The denervated rats exhibited attenuated exercise-induced increases in cutaneous heat loss, an exacerbated increase in MAP (Figure 4) and reduced spontaneous baroreflex sensitivity (Table 3). However, these changes did not result in impaired physical performance (Figure 3). Interestingly, denervation of the caudal artery only slightly affected thermal and cardiovascular regulation in rats under resting conditions (Figure 2) or during the post-exercise period (Figure 5). In this study, we also performed histological analyses of the caudal artery after denervation, revealing increases in the arterial wall-to-lumen ratio (Table 5). Finally, we provided evidence that CAD-induced vascular remodeling was associated with increased blood pressure and an attenuation of cutaneous heat loss during exercise, as evidenced by the negative correlations between the maximum T_SKIN_ attained or HL_SEN_ and the wall-to-lumen ratio and by the positive correlation between the maximum DAP attained and the wall-to-lumen ratio (Figure 7).

In resting rats, denervation of the caudal artery reduced and increased, respectively, the T_SKIN_ and T_CORE_ variability, thereby confirming our previous results (Lima et al., 2013). Based on a substantial amount of evidence, tail heat loss plays an important role in T_CORE_ control in rats; therefore, these changes in T_CORE_ and T_SKIN_ variability likely reflect an impaired fine-tuned control of T_CORE_ caused by the loss of sympathetic vasoconstrictor control of the tail vasomotor tone. However, despite the changes in temperature variability, average T_CORE_ and T_SKIN_ values were similar between the groups, as expected (Lima et al., 2013).

**Figure 8:**
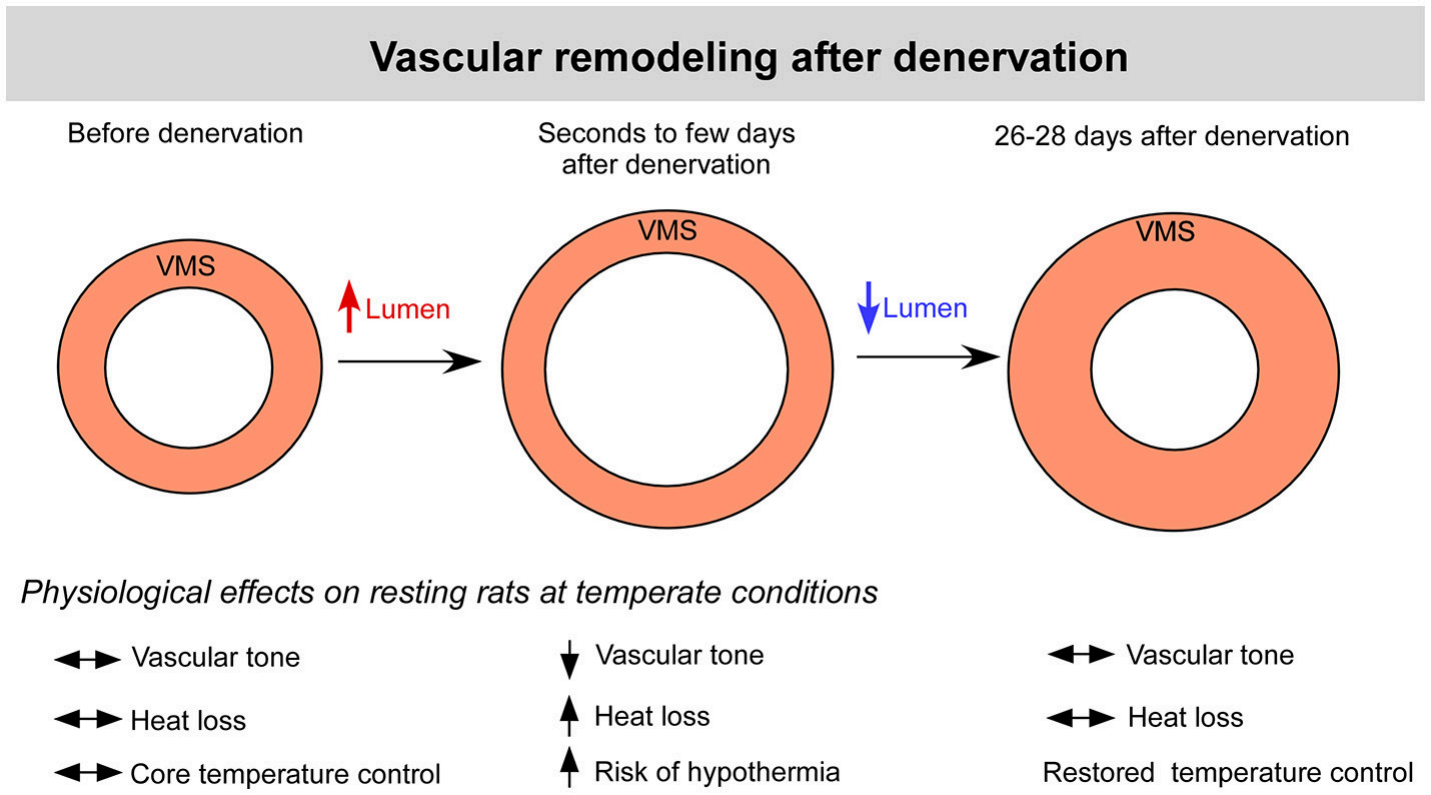
Changes in the caudal (tail) arteries of denervated rats. Within seconds of denervation of the caudal artery, intense local vasodilation is observed, enhancing the cutaneous heat loss and increasing the risk of hypothermia. This enhanced heat loss is likely counteracted by augmented heat production, an expensive metabolic condition that cannot be sustained for long periods. Therefore, functional (i.e., hypersensitivity to vasoconstrictor agents) and structural (i.e., vascular remodeling) adaptations develop to restore the vascular tone, lumen size and thermal balance. The vascular remodeling observed in the present study was characterized by an increase in the wall-to-lumen ratio, which seems to overcome the lack of sympathetic innervation under resting conditions but impair cutaneous heat loss during exercise. Legend: VSM, vascular smooth muscle.

In our previous study, marked vasodilation was observed a few seconds after phenol application on the proximal portion of the caudal artery (Lima et al., 2013); this vasodilation results from the chemical neurolysis of sympathetic innervation, which promotes the tonic vasoconstriction of cutaneous vessels (O'Leary et al., 1985). In this context, reestablishment of the baseline thermoregulation a few days after denervation (Yanagiya et al., 1999) would prevent a sustained hypothermic condition caused by the loss of sympathetic vasomotor tone and likely results from functional and structural adaptations in the caudal artery. The increase in vasomotor tone induced by the chronic absence of vascular innervation reflects the development of adaptations that favor vasoconstriction and occur subsequent to changes in vascular sensitivity to constrictor and dilator agents (produced locally or present in circulation). For instance, enhanced vasoconstriction is associated with vascular hypersensitivity following stimulation with several agents, such as norepinephrine, epinephrine, endothelin-1, vasopressin, and angiotensin II (Bevan and Tsuru, 1981; Taki et al., 2004; Kamikihara et al., 2007; Tripovic et al., 2010). Denervation induced structural adaptations in our study, as evidenced by hypertrophy of the vascular smooth muscle, without concomitant changes in the lumen size. Thus, vascular remodeling characterized by the concomitant increase in the wall-to-lumen ratio and the hypersensitivity to constrictor agents would help to restore cutaneous blood flow in the absence of sympathetic innervation to the tail (Figure 8).

The vascular remodeling observed in the denervated tail arteries may explain the slight but insignificant increase in MAP in resting rats. The increased artery wall thickness may have contributed to an increase in peripheral resistance that, in turn, would place a higher burden on the heart, as evidenced by the tendency for tachycardia to maintain blood perfusion pressure to peripheral tissues. Collectively, our findings showed a slight effect of CAD on cardiovascular control in resting rats 26–28 days after denervation without inducing hypertension. Nevertheless, we cannot disregard the possibility that CAD rats would have become hypertensive if the experiments were continued for a more prolonged period.

**Figure 9:**
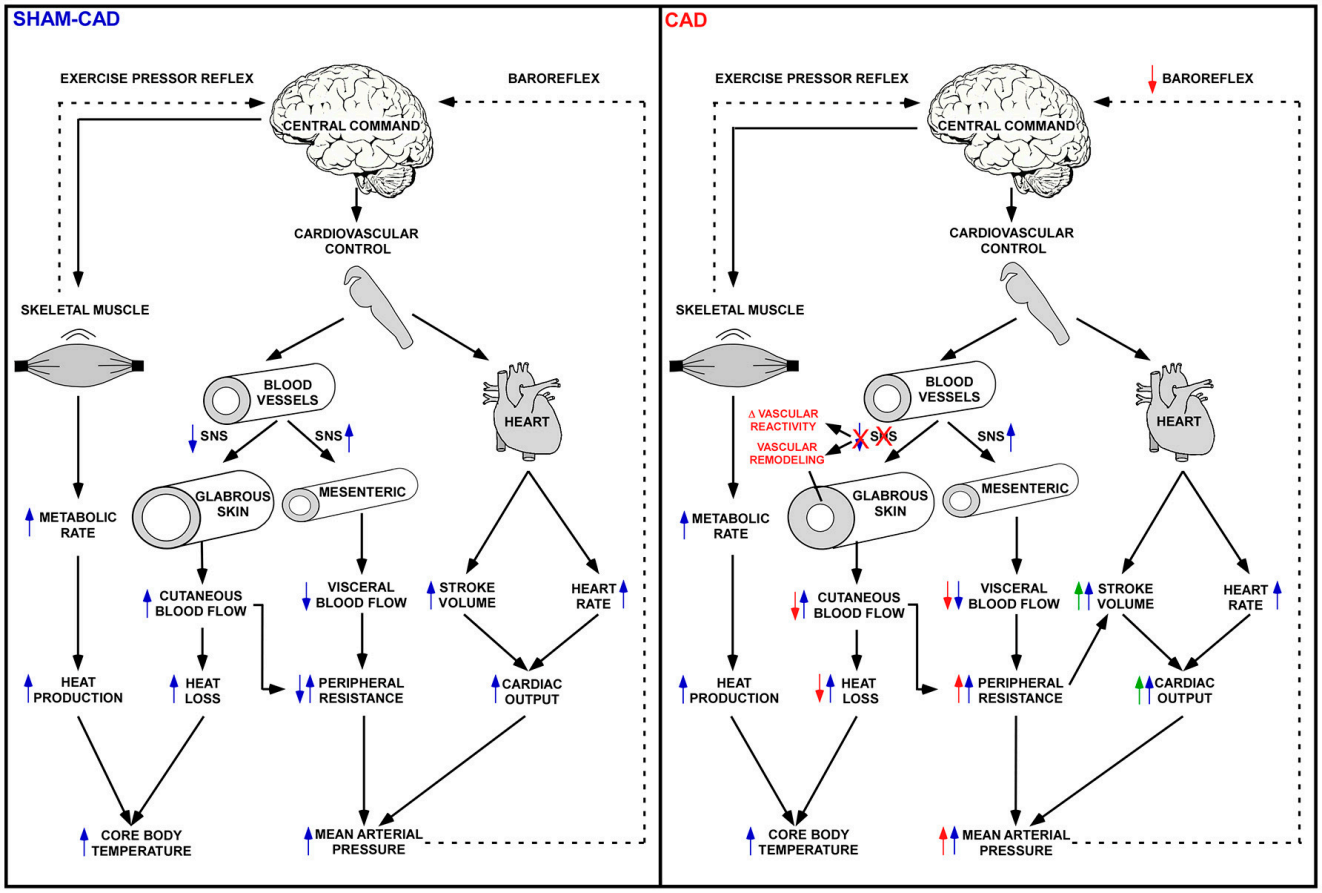
Relevance of cutaneous artery innervation to proper exercise-induced physiological adjustments. Skeletal muscle contraction increases the metabolic rate, and consequently, heat production. In a temperate environment, the increase in metabolic heat production is faster than the increase in heat loss (e.g., cutaneous vasodilation), thereby raising the core body temperature of running rats. Simultaneously, cardiac output is also augmented due to increases in the heart rate and stroke volume, resulting in increased arterial pressure. These cardiac changes are accompanied by blood flow redistribution, characterized by visceral and renal vasoconstriction and by skeletal muscle and skin vasodilation. Collectively, these adjustments supply oxygen and nutrients to the contracting muscles **(Left**: Sham-CAD). In the present study, the vascular adaptations that developed after cutaneous artery denervation impaired the cardiovascular and thermoregulatory adjustments induced by physical exercise (**Right**: CAD). The denervated rats exhibited lower skin temperatures while running, without concomitant changes in core temperature. They also had higher arterial pressures, likely to compensate for the enhanced peripheral resistance, which resulted in the attenuation of cutaneous heat loss. The exaggerated increase in blood pressure was not compensated by an attenuated increase in heart rate, indicating that caudal artery denervation impairs baroreflex function. Legend: The blue arrows indicate the expected exercise-induced cardiovascular and thermoregulatory adjustments (the blue arrows pointing up and down mean increase and reduction, respectively), whereas the red signals and text indicate changes induced by CAD (the red arrows pointing up and down mean exacerbated and attenuated responses, respectively). Finally, the green arrows indicate hypothetical changes induced by CAD (not investigated).

In exercising rats, the chronic absence of caudal artery innervation attenuated cutaneous heat loss and modified HL_SEN_, indicating that, in contrast to observations in resting rats, functional and structural adaptations following CAD affected tail heat loss during physical exertion. Based on the correlations shown in Figure 7, the increase in the wall-to-lumen ratio in CAD rats is associated with the attenuation of tail heat loss, the attenuation of HL_SEN_ and the exacerbation of DAP during exercise. Therefore, we propose that the increased wall-to-lumen ratio leads to increased vascular resistance in the caudal artery during exercise; thus, the rat experiences difficulty increasing local blood flow, consequently limiting tail heat loss (Figure 9).

Tail blood flow represents ~10% of the cardiac output in heat-stressed rats (Johansen, 1962). In exercising rats, reduced sympathetic activity to the tail artery has been hypothesized to cause vasodilation and increase tail heat loss; in humans, an initial withdrawal of vasoconstrictor activity in glabrous (i.e., non-hairy) skin occurs as the body heats, leading to an immediate increase in the level of blood flow (Johnson, 2010). Therefore, the inability of our CAD rats to increase tail heat loss during exercise cannot be explained by an impaired removal of sympathetic outflow because the sympathetic innervation in the tail was experimentally removed in these rats. The most likely explanation is that morphological and functional adaptations in the proximal portion of the caudal artery cause limited heat loss. As tail blood flow increases during exercise (as estimated by changes in tail-skin temperature; Tanaka et al., 1988; Wanner et al., 2015b), cutaneous vasodilation likely contributes to reduced peripheral resistance. Thus, attenuation of the expected exercise-induced vasodilation in association with the expected visceral and renal vasoconstriction (Rowell, 1974; Flaim et al., 1979) may limit the reduction in peripheral resistance and then exacerbate the increase in blood pressure in CAD rats (Figure 9). Notably, no study has determined the percentage of cardiac output represented by tail blood flow in rats running on a treadmill and the impact of tail-skin vasodilation on peripheral resistance.

Tail skin vasodilation is the main route for heat loss in exercising rats (Wilson et al., 1978; Shellock and Rubin, 1984). Surprisingly, a 40% reduction in tail heat loss caused no changes in T_CORE_ and thus did not impair running performance. Because this substantial attenuation of tail heat loss did not exacerbate T_CORE_, other pathways, which are often disregarded, likely facilitated heat exchange with the surrounding environment. Evaporative loss by breathing, ear vasodilation, and vasodilation of the medial and distal portions of the caudal artery (tail regions unaffected by our denervation procedure) are possible compensatory pathways. Supporting the first of these hypotheses, a previous human study demonstrated that individuals who showed lower skin vasodilation during exercise exhibited greater hyperthermia-induced hyperventilation (Hayashi et al., 2009). Another relevant issue is that our exercise experiments were performed under temperate conditions that did not cause high thermoregulatory strain. In contrast, if the experiments were performed in a hot environment, these alternative heat loss pathways would likely not compensate for the impaired tail heat loss, reducing physical performance and increasing the risk of hyperthermic damage in CAD rats.

In exercising CAD rats, cutaneous heat loss dysfunction was accompanied by an exacerbation of the increase in MAP (Figure 4). Consistent with their exacerbated MAP response during exercise, CAD rats exhibited SAP recordings with higher standard variation and variance coefficient values, indicating impaired fine control of blood pressure. Additionally, these rats displayed higher LF power in the SAP variability, potentially indicating enhanced lumbar sympathetic nerve activity (Waki et al., 2006), which modulates the sympathetic vasomotor tone of the rat tail, as revealed by the occurrence of cutaneous vasoconstriction after stimulation of lumbar segments (Rathner and McAllen, 1998). However, this mechanism does not seem to be a reasonable explanation for the present data, as the sympathetic innervation to the tail was denervated in these rats. A putative mechanism to explain the greater LF power in CAD rats is a stiffer arterial tree. This hypothesis is supported by previous observations that the LF power of the SAP variability is associated with reduced arterial distensibility (i.e., greater arterial stiffness) in spontaneously hypertensive rats (Dabiré et al., 2002). Interestingly, the altered LF power was only observed during exercise, a condition in which greater strain is placed on physiological systems, thereby facilitating the disclosure of cardiovascular control impairments.

One of our hypotheses was that impaired vasodilation in CAD rats would increase peripheral resistance and then place a burden on the cardiovascular system. However, our data do not support this hypothesis, as the increase in blood pressure in the CAD rats compared to that in the Sham-CAD rats occurred before the CAD-induced changes in T_SKIN_. Two non-mutually exclusive mechanisms may explain this observation. First, as increased sensitivity to catecholamine is a classic adaptation developed after tail artery denervation, we postulate that the accelerated increase in blood pressure is induced by greater tail sensitivity to catecholamines of neural and humoral origins (Kamikihara et al., 2007; Tripovic et al., 2010), the levels of which increase immediately after exercise initiation, thereby promoting peripheral resistance. Second, as rats began to exercise, the stroke volume, and consequently, cardiac output may have increased because of greater cardiac sympathetic outflow to overcome increased peripheral resistance in CAD rats; the latter explanation seems unlikely since autonomic cardiac control during exercise was not affected by denervation. Although the underlying mechanism remains unclear, the observation that changes in arterial pressure preceded changes in thermoregulation indicates that impaired tail vasodilation may have contributed, but was not the triggering factor, to the exaggerated arterial pressure during exercise.

Despite the exaggerated increase in MAP, HR was not affected by CAD, suggesting an impaired baroreflex sensitivity in these rats. Indeed, the evaluation of spontaneous baroreflex sensitivity confirmed our hypothesis; CAD impaired sensitivity during exercise. Supporting the lack of inter-group differences in HR during exercise, the spectral analysis of the pulse interval did not differ between CAD and Sham-CAD rats. Interestingly, the lack of CAD-mediated changes in running-induced tachycardia is unexpected because resting CAD rats displayed a trend toward higher HR values.

CAD rats exhibited a transient increase in T_SKIN_ during the initial 10-min post-exercise period. Notably, CAD rats were more reactive to vasoconstrictor agents and likely displayed greater vasodilation than Sham-CAD rats in response to the suppression of these agents, as expected with exercise termination. Another explanation is that CAD rats require a longer period to achieve maximum T_SKIN_ values during exercise. This hypothesis corroborates our previous findings that CAD delays the increase in T_SKIN_ but does not change the maximum T_SKIN_ value attained in rats that were passively exposed to heat (Lima et al., 2013). Similarly, although the rat's tail was still sensitive to cold, despite the disconnection from supraspinal centers, this local cold-induced vasoconstriction effect was slower in lesioned rats (Kalincik et al., 2009). Finally, the increase in T_SKIN_ of CAD rats at the beginning of the post-exercise period may have reduced peripheral resistance, thereby preventing the observation of inter-group differences in cardiovascular parameters.

The hypothesis that morphological adaptations to the cutaneous denervated vasculature are associated with altered cardiovascular and thermoregulatory adjustments during exercise was confirmed in the present study. However, the adaptations observed following sympathetic denervation of the tail occurred in the opposite direction from those observed in the denervated ear arteries of “young adult” rabbits, in whom sympathetic denervation reduced the total wall thickness and the cross-sectional area of the tunica media (Bevan, 1975; Bevan and Tsuru, 1981). These inter-study differences may be explained by methodological differences, such as the animal species, the denervated site, the denervation technique and the time after denervation at which the experiments were performed. Here, we have shown for the first time that in addition to promoting changes in vascular reactivity, CAD promotes vascular remodeling in rats that are observed ~1 month after the procedure. The occurrence of this remodeling was further supported by the observation that cutaneous HL_SEN_ was reduced in CAD rats, indicating that changes in peripheral control of the vasomotor tone occurred.

Aerobic performance was reduced during the second incremental exercise relative to the first one irrespective of the experimental group (Figure 3). This reduction in performance may be partially explained by the body mass gain of the rats (~80 g) during the 26-day interval between the two exercises, as evidenced by the negative correlation between the time to fatigue and body mass. The influence of body mass on performance measured on the treadmill is explained by the fact the rat must support and transport its body while running. Our data agree with previous investigations in which untrained rats tested regularly in 4-week intervals exhibited body mass gains and shorter times to fatigue during an incremental exercise similar to the one used here (Teixeira-Coelho et al., 2017). Another likely explanation for the reduced performance in the second exercise is the fact that the rats were not additionally familiarized to the experimental setup between the two exercises; this 26-day period was likely long enough to reduce familiarization with running on the treadmill, thereby accelerating fatigue.

In conclusion, CAD attenuated and enhanced exercise-induced increases in cutaneous heat loss and blood pressure, respectively. Moreover, the denervation procedure promoted vascular remodeling in the caudal artery, as evidenced by the increased arterial wall thickness and the wall-to-lumen ratio. Notably, CAD-induced vascular remodeling was associated with altered physiological responses during exercise, suggesting that vascular remodeling at least partially contributes to the attenuated cutaneous heat loss and enhanced blood pressure observed in denervated rats during exercise.

### Physiological relevance

Understanding thermoregulatory and cardiovascular system reactions after an abrupt and sustained cutaneous vascular innervation injury (Lepori et al., 1999; Charkoudian et al., 2002; Yeoh et al., 2004) is essential for the treatment of some diseases. For example, individuals with Parkinson's disease exhibit disrupted cutaneous vascular innervation (Navarro-Otano et al., 2015) that is associated with abnormal skin thermal responses during cold stress (Antonio-Rubio et al., 2015). Local sympathetic denervation is also observed in patients with painful diabetic neuropathy (Tack et al., 2002); indeed, individuals with type 1 and type 2 diabetes mellitus, particularly patients with neuropathy, have impaired abilities to maintain T_CORE_ during either hot or cold thermal stress (Kenny et al., 2016). Here, the application of phenol to the caudal artery was used as a model to induce an abrupt and sustained local denervation of the cutaneous vasculature in rats (Lima et al., 2013). We revealed the importance of cutaneous vascular innervation integrity in thermal and cardiovascular control in stress-challenged rats.

## Author contributions

Conceived and designed the research: MM-L, WP, IF, CC, NL, and SW; Performed the experiments: MM-L, WP, IF, and JJ-S; Analyzed the data: MM-L, WP, IF, JJ-S, AF, CC, NL, and SW; Interpreted the experimental results: MM-L, WP, IF, AF, CC, NL, and SW; Prepared the figures: MM-L and SW; Drafted the manuscript: MM-L and SW; Edited and revised the manuscript: MM-L, WP, CC, and SW; Approved the final version of the manuscript: MM-L, WP, IF, JJ-S, AF, CC, NL, and SW.

### Conflict of interest statement

The authors declare that the research was conducted in the absence of any commercial or financial relationships that could be construed as a potential conflict of interest.
